# Densely Packed Li‐Metal Growth on Anodeless Electrodes by Li^+^‐Flux Control in Space‐Confined Narrow Gap of Stratified Carbon Pack for High‐Performance Li‐Metal Batteries

**DOI:** 10.1002/advs.202205328

**Published:** 2022-11-24

**Authors:** Jong Ho Won, Woo Hyeong Sim, Donghyoung Kim, Hyung Mo Jeong

**Affiliations:** ^1^ Department of Chemistry Kookmin University 77 Jeongneung‐ro, Seongbuk‐gu Seoul 02707 Republic of Korea; ^2^ School of Mechanical Engineering and Department of Smart Fab. Technology Sungkyunkwan University 2066 Seobu‐ro Suwon 16419 Republic of Korea

**Keywords:** anode‐free, dendrite‐free lithium growth, lithium metal batteries, space confinement effect, stratified carbon

## Abstract

Lithium (Li) is the “holy grail” for satisfying the increasing energy demand. This is because of its high theoretical capacity and low potential. Although Li is considered as a potential anode material, dendritic Li growth and the limited electrochemical properties continue to hinder its practical application. Structure‐based self lithium ion (Li^+^) concentrating electrodes with high capacity and uniform Li^+^‐flux are recommended to overcome these shortcomings of Li. However, recent studies have been limited to structural perspectives. In addition, the electrokinetic principle of electrode materials remains a challenge. Herein, the space‐confinement‐based strategy is suggested for condensed Li^+^‐flux control in nanoscaled slit spaces that induce the dense Li growth on an anodeless electrode by using the stratified carbon pack (SCP). The micro/mesoporous slits of the SCP concentrate the electric field, which is strengthened by the space‐confined electric field focusing, resulting in the accumulation of Li^+^‐flux in the host. The accumulated Li^+^ in host sites enables a uniform Li deposition with high capacity at high current density stably. Furthermore, SCPs have great compatibility with LiNi_0.8_Co_0.1_Mn_0.1_O_2_ (NCM811) cathode, representing the outstanding full cell performance with Li deposited electrode which show the high specific of 115 mAh g^−1^ at 4 C during 350 cycles.

## Graphical abstract

The stratified carbon pack (SCP) introduces electric double layer focusing induced litihium ions accumulation from structured features of SCP with nano‐scaled space, thus enabling dendrite‐free growth with a densely packed deposition of lithium, and the durable operation over 250 h (at 4.0 mA cm^−2^) and over 350 cycles (at 4.0 C) in a symmetric and LiNi_0.8_Co_0.1_Mn_0.1_O_2_ full‐cell configuration, respectively.

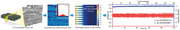

## 1 Introduction

Advanced rechargeable batteries are required to address energy issues and thereby satisfy the increasing demand for energy density and power density for energy storage technology (such as electric vehicles and energy storage systems).^[^ ^1^, ^2^, ^3^ ^]^ From this perspective, lithium (Li) is considered as one of the highly potential candidates. This is owing to its low redox potential (−3.04 V versus standard hydrogen electrode) and high theoretical capacity (3860 mAh g^−1^), which yield a high power and energy density.^[^ ^4^, ^5^, ^6^ ^]^ Although Li is the “holy grail” with regard to Li ion (Li^+^)‐based batteries, the uncontrollable characteristic of Li causes certain issues such as dendritic Li growth and extreme volume expansion. These issues hinder the practical application of Li batteries.^[^ ^7^ ^]^ The rough surface of copper electrodes with disordered electrode tips focus Li^+^, which causes uncontrollable Li growth by the tip effect. This unmanageable behavior of Li^+^ causes the non‐uniform formation of the solid electrolyte interphase (SEI) and dendritic Li growth. These features result in an inferior electrochemical operation involving safety hazards. In a general Li battery system, Li^+^ is more concentrated in the electrode tip. This induces a non‐uniform flux of Li^+^.^[^ ^8^ ^]^ Moreover, extreme volume expansion during Li plating/stripping is accompanied by undesirable side reaction and non‐uniform Li^+^ flux. This induces the loss of Li^+^ and formation of inactive Li.^[^ ^9^, ^10^ ^]^

To address the unstable operation and limited energy density of previous Li‐metal electrodes, advanced host electrode materials that overcome the unbalance of local current density and Li^+^ flux and enables stable Li^+^ control are recommended.^[^ ^11^, ^12^, ^13^, ^14^, ^15^ ^]^ This locally high current density and Li^+^ flux at a rough electrode promotes the non‐uniform reaction and fast degradation of Coulombic efficiency (C.E.). This, in turn, leads to severe disadvantages for practical usage.^[^ ^16^, ^17^ ^]^ To date, several studies have recommended certain host materials such as 3D porous structured host materials (e.g., structured carbon and Cu electrode) to control Li^+^ by regulating the local current and ion flux.^[^ ^18^, ^19^, ^20^, ^21^, ^22^, ^23^ ^]^ Among these materials, 3D structured carbon electrodes have been proposed for regulating Li behavior because of its manageable process and high electrochemical stability for electrochemical reactions.^[^ ^20^, ^21^ ^]^ From this perspective, structure‐based spontaneous Li^+^ preference electrodes without other elemental addition have been examined to maximize the advantages of own electrodes. Liu et al.^[^ ^9^ ^]^ demonstrated that a modified electrode with nanochannels generates relatively identical Li^+^ flux in each channel. This results in enhanced C.E. and lifespan. Wang et al.^[^ ^16^ ^]^ elucidated that a current collector with channels having an appropriate size reduces the local current density and suppresses the dendritic Li growth. Niu et al.^[^ ^12^ ^]^ investigated a Li—C anode. It comprised a mesopore/cavity with an amine functional group that offers a reliable Li deposition site by inducing Li deposition into the cavity first. From an experimental perspective, these studies demonstrated that structural properties can activate the self Li^+^ concentration and thereby cause uniform Li growth by locally controlled Li^+^ behavior. However, an additional material portion of electrodes induce the reduction of energy densities although functional materials can control the Li^+^ and stable Li growth. Although the structural properties have been indicated as the effective factors for controlling the Li growth to a certain extent, further approaches with regard to experimental and mechanism aspects are necessary to retain the strategy for both sufficient Li storage by densely packed Li growth and a specific structural design principle of electrode materials for Li^+^ flux control. Advanced structural approaches toward electrode materials capable of controlling Li^+^ and increasing energy density are required by densely packed Li‐metal growth to realize effective anodeless electrodes.

In terms of the electrokinetic phenomena with porous structured media, an activated ion is confined by sub‐nanoscaled space in local electrode sites with an electric field. Furthermore, an electrochemical reaction of this ion can be facilitated.^[^ ^13^ ^]^ That is, a strong inner electric field generated by an overlapped electric double layer (EDL) promotes electrochemical reaction in the electrode space through electro‐osmosis during ion kinetic behaviors.^[^ ^13^ ^]^ In this respect, conventional graphite electrodes cause sluggish reactions owing to the atomic‐scale gap of carbon layers which induce the strong interaction between carbon layers.^[^ ^24^ ^]^ These structural features of graphite electrode induce the intercalative Li storage with limited energy density and slow kinetics, and cannot handle the Li‐metal for improved battery performance. It should be expected that the critical size‐range of space is required to enable formation of uniform Li^+^ flux for Li‐metal accommodation in electrode materials. From the structural perspective, if an electrode material with appropriate range of narrow slits is used in a battery system, Li^+^ is concentrated in the slits thermodynamically. This induces a stable electrochemical reaction.^[^ ^12^ ^]^ The adaption of this space feature to electrode materials results in a strong interaction with Li^+^. Consequently, a narrow gap is essential to effectively utilize the Li^+^ flux control on electrodes for a stable and high performance operation of Li electrodes. Locally condensed Li^+^ in narrow spaces induce dendrite‐free Li growth with a rapid storage reaction. This enables the Li growth control to achieve densely packed Li storage for the Li storage system with anodeless electrodes. As the porous structure of electrode materials are confined to the nanoscale, ions are attracted into the pores spontaneously owing to EDL and electro‐osmosis reinforcement.^[^ ^25^ ^]^ When nano‐ and sub‐micron‐sized spaces exist in the electrode structure, the Li^+^ transport in the electrode structure is promoted by enhancing the electrokinetic phenomena.^[^ ^26^ ^]^ Therefore, structural factors of electrode materials with a nano‐ or sub‐micron‐slit distance are required to achieve the Li^+^ control behavior and an enhanced electrochemical reaction.

In this study, local Li^+^‐flux condensation that induces densely packed Li growth was established by the nanoscaled slit spaces in the stratified carbon packs (SCPs), thereby realizing the anodeless electrodes for Li batteries. From the self‐assembly‐based synthetic method of SCP, each carbon layer is packaged with nanoscaled slits, which attract Li^+^ by EDL overlapping in confining spaces during Li stripping/plating. The SCP is synthesized by a facile method using graphene with adhesive and additional heat treatment. The process generates suitably sized slits and nitrogen sites. The Li^+^ is attracted into the space by the Li^+^ preference feature of SCPs. This generates a locally concentrated Li^+^ flux in slits, which results in densely packed Li inside slits by hindering the dendrite growth. The Li growth behavior on SCP electrodes is controlled effectively by the selective condensation of Li^+^ flux in confined spaces. Consequently, the cell configuration with SCP electrodes exhibits a high electrochemical performance during stable operation at an exceptionally high current density with a high areal capacity. The SCP electrodes with controlled Li deposition enable the construction of a full‐cell configuration with commercially available cathode materials LiNi_0.8_Co_0.1_Mn_0.1_O_2_ (NCM811). These exhibit a capacity retention of almost 100% at a scan rate of 4 C.

## 2 Results and Discussion

### 2.1 Li Growth Behavior on Electrodes with Space‐Confined Structure

**Figure 1:**
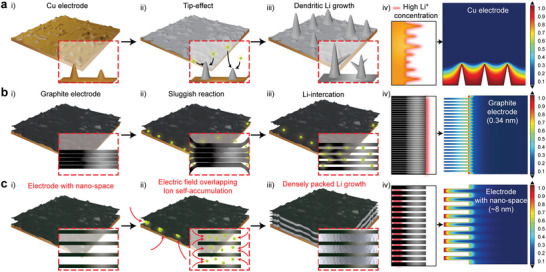
Schematic diagram of Li storage mechanism with calculated Li ion concentration contribution according to electrode structure such as a) Cu electrode, b) graphite electrode, and c) electrode with nano‐space.

With regard to the electrode structure and inner space, the numerous tips and rough structure of a Cu electrode cause severe dendritic Li growth and a low lithium storage capability. These features of the electrode cause Li^+^ to be concentrated at the rough electrode tip. This, in turn, causes dendritic Li growth (**Figure** **1**a,i–iii). The calculation results obtained using the COMSOL multiphysics data also indicate that a non‐uniform Li^+^ concentration is induced by the coarse structure of the electrode. (Figure 1a‐iv). These results imply that Li^+^ behavior regulation and reliable Li growth can be achieved by controlling the electrode structure. Commonly used graphite electrodes have exceptionally narrow gaps by the interaction of carbon layers. Consequently, Li^+^ cannot permeate straightforwardly into the graphite electrode and therefore accumulated on the surface of the graphite electrode during charging (Figure 1b,i–iii). In the calculation data, a conventional graphite electrode with a narrow gap (which influences the Li^+^ intercalation) reveals the accumulation of Li^+^ on the graphite surface (Figure 1b‐iv). Therefore, the formation of a specific structure capable of controlling Li^+^ is important for reliable and robust Li deposition. From the above perspective, an electrode material with nanospaces (which enables electric field overlapping) causes ion self‐accumulation. (Figure 1c,i–ii) This unique structured electrode for Li behavior controls and guides the Li^+^ flux condensation inducing the densely packed Li growth. Consequently, a narrow gap is important for contributing to high‐end energy storage devices by reinforcing stable Li deposition (Figure 1c‐iii). In the COMSOL multiphysics data, Li^+^ concentration focusing into an 8 nm‐scaled carbon slit is verified except for the sluggish intercalation reaction (Figure 1c‐iv). In addition, Li^+^ accumulation into the electrode is confirmed at 2 nm‐scaled carbon slits (Figure S1a, Supporting Information), demonstrating Li^+^ behavior is controlled by adjusting gap spacing of an electrode below a certain scale of distance. On the other hand, Li^+^ are concentrated on the electrode surface rather than inside an electrode at a relatively wide gap distance of 15 nm (Figure S1b, Supporting Information). These simulation and theoretical results reflect the importance of Li^+^ behavior control induced by the slit structure for densely packed Li growth and high battery performance.

### 2.2 Fabrication of Anodeless Electrodes with Space‐Confined Narrow Gap

**Figure 2:**
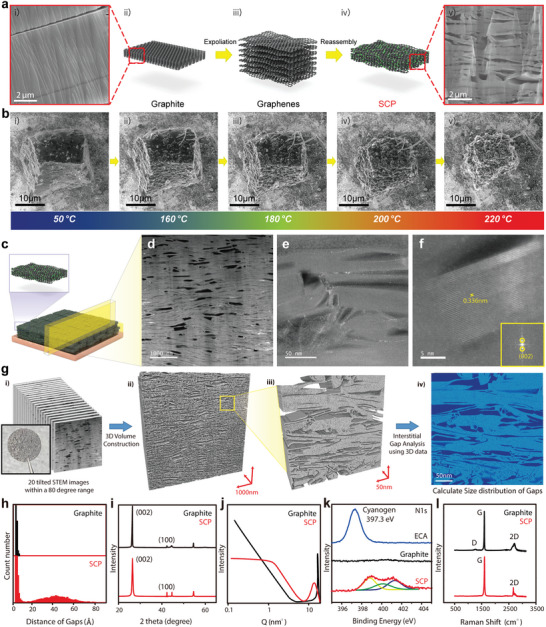
Fabrication and characterization of SCPs. a) Schematic illustration and scanning electron microscopy (SEM) images according to synthesis process of SCPs. b) The SEM images during annealing the SCPs. The SCP is shrunk after heat treatment, remaining lithiophilic nitrogen (N) component. c–f) Cross‐sectional scanning transmission electron microscopy (STEM) image of the synthesized SCPs. g) The flow chart for the construction of a 3D volume using 20 tilted STEM images within an 80° range, and measurement of the spacing distribution based on the 3D volume. h) Gap distribution of graphite and SCPs obtained from 3D volume based calculation. i) The X‐ray diffraction (XRD) spectra of graphite and SCPs. j) The small‐angle X‐ray scattering (SAXS) analysis for graphite and SCPs. k) The chemical composition analysis of polymers, graphene, and SCPs by X‐ray photoelectron spectroscopy (XPS). l) The Raman spectra of graphite and SCPs.

Following the material design strategy for Li^+^ flux control in a confined structure for an anodeless electrode, the SCP with a specific range of carbon slit has been recommended by re‐assembling the individual 2D carbon materials. As shown in the schematic diagram in **Figure** **2**a, the SCP is synthesized by the process of graphene re‐assembly. As is evident from the scanning electron microscope (SEM) image (Figure 2a‐i,v), SCP has micro‐nanoscale slits and pores, whereas graphite does not have additional space. SCP is synthesized by a facile self‐assembly packaging method,^[^ ^27^ ^]^ as shown in Figure 2b and Figure S2, Supporting Information. The polymer used for self‐assembly is an adhesive called ethyl cyanoacrylate. It is rich in nitrogen and can be removed completely by heat treatment below 200 °C. An environmental SEM analysis was conducted to observe the synthetic procedure during heat treatment (Figure 2b). At the stage of the graphene–adhesive mixture (Figure S2b, Supporting Information), the adhesive reacts with moisture and anions located on the graphene surface to polymerize and harden highly rapidly within graphene.^[^ ^27^ ^]^ When a mixture of graphene and adhesive is placed in an electric furnace under vacuum and heated, the adhesive begins to attain a flexible and sticky state (Figure 2b‐i and Figure S2c, Supporting Information). When the heat treatment temperature exceeds 160 °C (Figure 2b,ii–v), the mixture of adhesive and graphene starts to shrink because the adhesive begins to decompose rapidly. The sticky adhesive placed between graphenes holds these close together while these are removed by heat treatment (Figure S2d, Supporting Information). At a temperature of over 200 °C, the graphene lamination is completed, and the graphene forms a stacked structure close to each other by decomposing the adhesive completely (Figure S2e, Supporting Information). These results are also supported by the thermogravimetric analysis data in Figure S3a, Supporting Information, and the differential scanning calorimeter analysis data in Figure S3b, Supporting Information.^[^ ^28^ ^]^

SEM and transmission electron microscope (TEM) analyses were conducted to compare the structural difference of the general graphite and as‐synthesized SCPs. Figure 2c–f shows a schematic illustration and cross‐sectional scanning transmission electron microscope (STEM) images of SCPs. SCPs have a large number of slits packaged at the nanoscale. These planar spaces are constructed to accommodate Li^+^ by concentrating the electric field. On the contrary, graphite has a lattice distance of about 0.34 nm without nanoscale slit spaces for extra Li storage (Figure S4, Supporting Information). As‐prepared graphite electrodes have a spherical shape and densely packed structure without a pore (Figure S5a, Supporting Information), whereas the SCPs have a plate‐like structure rich in slits and pores (Figure S5b, Supporting Information). Similar to SEM analysis, a stacked graphene layer is clearly observed by TEM analysis (Figure S6, Supporting Information). For further information on space, the gap distribution was calculated by constructing the 3D volume within an 80° range using 20 tilted scanning transmission electron microscope (STEM) images^[^ ^29^, ^30^ ^]^ (Figure 2g). Based on the calculation data, SCPs have a planar distance similar to that of graphite. However, the SCP also has a mesoscale space and cavity in packaged structures.^[^ ^29^, ^30^ ^]^ Figure 2h shows a distinct difference wherein SCP has a larger number of narrow slits than graphite, and SCP has a mesopore and slit of sizes 20–80 Å. This porous structure of SCP yields the high and stable Li reserve property by concentrating Li^+^ into the slit with a rapid Li storage reaction.^[^ ^31^, ^32^ ^]^ This corresponds with the theoretical analysis presented in Figure 1. These features are likely to generate the thermodynamic self‐smoothing characteristic of Li in the cavity and space, thereby resulting in dendrite‐free Li growth and remarkable operation. The structure of SCPs and graphite are compared by X‐ray diffraction (XRD) analysis as well (Figure 2i). Comparing the full width at half maximum (FWHM) of the (100) position pick of graphite and SCP, it is verified that the SCP derived from graphene has a long crystal structure in the planar direction compared with graphite. This indicates that planar and wide graphene is stacked flawlessly in parallel during the SCP synthesis.^[^ ^33^, ^34^ ^]^ In addition, comparing the full width at half maximum (FWHM) of the (002) position pick of graphite and SCP, it is verified that the vertical grain size of SCP is shorter than that of graphite. This is owing to the nanoscale slit generated inside the SCPs.^[^ ^33^, ^34^ ^]^ The results of a small‐angle X‐ray scattering (SAXS) analysis (Figure 2j) reveals that sub‐nanoscale and macro‐scale pores are mainly observed in graphite, whereas many nanoscale micropores are observed in SCP owing to the internal nanoscale slits.^[^ ^35^ ^]^ Thus, the XRD analysis results effectively reveal the structural properties of SCPs derived from the synthesis method compared with graphite. The pore distribution results obtained by argon adsorption (see Figure S7, Supporting Information) verify that SCPs have nanosized micropores unlike graphite.^[^ ^36^ ^]^ X‐ray photoelectron spectroscopy (XPS) analysis was conducted to compare the chemical bonding states of SCPs and graphite (Figure 2k). A cyanogen‐based adhesive was used to synthesize the SCP. The XPS data at N 1s region shows the cyanogen bonding peaks. Pyrrolic, pyridinic, and graphitic N bonding were observed after the synthesis of SCP. The N‐configuration in electrode materials is known to have stronger binding energy with Li^+^ than the C or Cu component, inducing the controlled Li growth and stable battery operation.^[^ ^37^ ^]^ This implies that the nitrogen atom of cyanogen is partially doped on the plane of SCPs during heat treatment.^[^ ^38^, ^39^ ^]^ The results of Raman spectroscopy tests on SCPs and graphite reveal that both the materials show strong G‐pick, whereas D‐pick is almost absent. This indicates that both the materials consist of a continuous hexagonal carbon structure without defects. However, the two materials show a difference in the shape of the 2D pick. It can be observed that the SCPs contain more graphene‐like structures with a small thickness owing to the 2D pick appearing at 2680 cm^−3^.^[^ ^40^, ^41^ ^]^

### 2.3 Effects of Space‐Confined Narrow Gap on Li Growth Behavior

**Figure 3:**
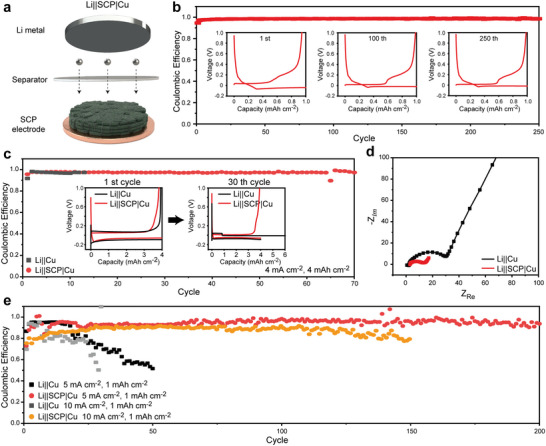
Electrochemical performance of Li||SCP|Cu. a) Schematic of Li||SCP|Cu. b) Coulombic efficiency (C. E.) of SCP half‐cell at a current density of 1.0 mA cm^−2^, with an areal capacity of 1.0 mAh cm^−2^. Inner profile shows the voltage hysteresis at 1st, 100th, and 250th cycles. c) Expanded property of Li||SCP|Cu at current density of 4.0 mA cm^−2^ with an areal capacity of 4.0 mAh cm^−2^. The Li||Cu shows the short‐circuit voltage behavior after 30 cycles. d) The resistance comparison of Li||Cu and Li||SCP|Cu by electrochemical impedance spectra (EIS). e) C. E. comparison with various current densities at 5.0 and 1.0 mA cm^−2^, with an areal capacity of 1.0 mAh cm^−2^.

Half‐cell configurations were evaluated at various current densities and areal capacities to verify the stable operation of SCP electrodes with controlled Li growth. A schematic illustration of the configured cell is shown in **Figure** **3**a. The half‐cell configuration with SCP electrode (Li||SCP|Cu) is operated with a high C.E. (>95%) and long cycle life (>250 cycles) at a current density of 1.0 mA cm^−2^ with an areal capacity of 1.0 mAh cm^−2^ (Figure 3b). The inlet profile shows the two plat region, showing the Li storage mechanism of Li intercalation and deposition. To compare the acceptable Li capacities of SCP electrodes, a half‐cell test is conducted at a relatively high current density of 4.0 mA cm^−2^ with a high areal capacity of 4.0 mAh cm^−2^ (Figure 3c). At a high Li acceptance level, Li||SCP|Cu accepts a relatively large amount of Li^+^ during 70 cycles without short‐circuit, whereas a half‐cell with a blank Cu electrode (Li||Cu) accepts a Li storage of an amount that is significantly less than that for Li||SCP|Cu. As shown in the inlet profile, Li||Cu shows the plat profile owing to the internal short circuit within 10 cycles. In Figure 3d, Li||Cu has a higher electrical resistance than Li||SCP|Cu. This indicates that Li||SCP|Cu forms a stable interphase with Li‐metal. Current densities of 5.0 and 10.0 mA cm^−2^ were applied to half‐cells with an areal capacity of 1.0 mAh cm^−2^ to demonstrate the stability under a severe current density condition (Figure 3e). At relatively high current densities, Li||SCP|Cu shows a stable operation with a high C.E., whereas Li||Cu shows a rapid reduction in C.E. within 50 cycles. In addition, half‐cell configuration with graphite and graphene electrodes show the relatively poor C.E. and cyclability at various electrochemical conditions (Figure S8, Supporting Information). Based on an electrochemical analysis and the Li deposition behavior, a uniform Li deposition and the self‐smoothing phenomenon of the electric field and EDL concentration in the slit structure of SCPs enable the improvement in electrochemical operation without other additional active materials.^[^ ^13^ ^]^ To support the practical understanding of EDL concentration according to the slit distance, cyclic voltammetry (CV) method is applied to graphite and SCP electrodes at a scan rate of 50 mV s^−1^ with a voltage range of 0.5–1.0 V (Figure S9, Supporting Information). As shown in the CV profile, SCP has a wider hysteresis loop than graphite electrodes, indicating high‐level Li^+^ accumulation is accommodated in the slits of SCPs.

**Figure 4:**
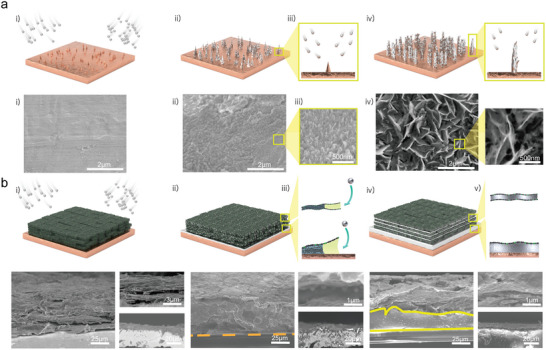
Schematic diagram and SEM images of bare electrode and SCP electrodes according to Li deposition at various steps. a) Dendritic Li growth on bare Cu electrodes and b) densely packed Li growth on SCP electrodes without Li dendrite.

Compressed Li growth with dendrite‐free behavior is necessary to accommodate the relatively high areal capacity of 4.0 mAh cm^−2^ for practical batteries. In addition, the battery operation condition with a high current density‐induced dendritic Li growth and high overpotential causes a rapid reduction in battery performance. To identify the principles of Li‐metal growth on SCP electrodes, by which SCP induces a reliable electrochemical performance, SEM analysis was conducted to confirm the Li deposition. In Li||Cu (**Figure** **4**a), Li nucleation and growth occurs by a tip‐induced mechanism. In general, more electrons are accumulated on a rough tip. This results in the non‐uniformity of the electric field. A non‐uniform electric field is known to induce a heterogeneous Li^+^ flux and Li deposition. SEM images show dendritic Li growth according to Li deposition. In the case of Li||SCP|Cu (Figure 4b), SCP enables Li^+^ accumulation and stable Li deposition into the narrow gap of the SCP structure by Li^+^ flux control and the ion confinement effect with EDL focusing.^[^ ^13^ ^]^ As shown in the SEM images, Li^+^ is first pre‐deposited into the slit. Densely packed Li growth is verified during Li plating. In addition to the calculated Li^+^ concentration gradient (Figure 1c), this controlled Li^+^ behavior on the SCP electrode demonstrate that dendrite‐free and reliable Li plating/stripping can be achieved by the electrode structure and space controlling. With these unique structural characteristics of SCP, the lithiophilic N elements in SCP support the amplification of Li^+^ attraction during Li deposition. Li deposition behavior according to the amount of deposited Li was observed by cross‐sectional SEM analysis for a more detailed explanation of the Li^+^ concentration on the SCP electrode (Figure S10, Supporting Information). During Li^+^ accumulation, Li is deposited into the SCP slit with dendrite‐free Li behavior notwithstanding the Li deposition rate. From the SEM and simulation analysis, the behavior of Li^+^ can be regulated by controlling the internal space of the electrode. Thus, these results reflect that the stable stripping/plating characteristic because of the structure‐based Li^+^ behavior control enables the attainment of a realistic energy density and battery stability for a high‐end battery.

### 2.4 Electrochemical Performances of Cell Configuration with Anodeless Electrodes Consisting of SCPs

**Figure 5:**
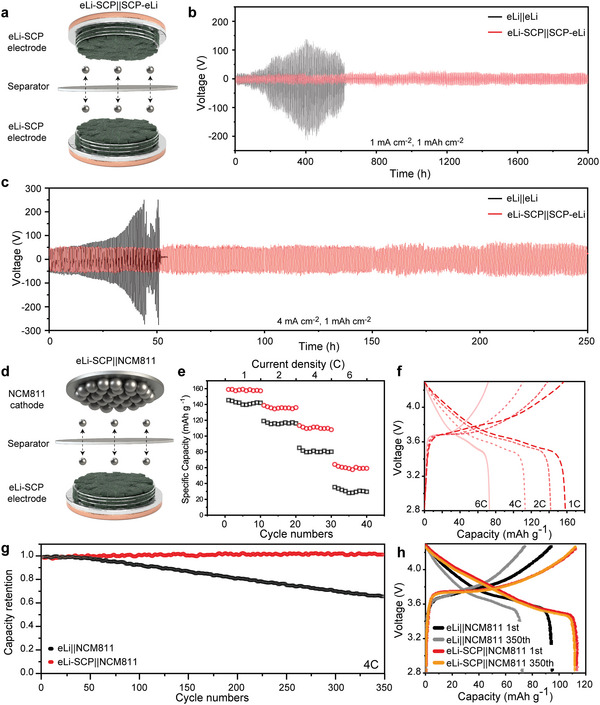
Practical batteries evaluation of symmetric configuration and asymmetric configuration with SCP electrodes. a) Schematic of eLi‐SCP||SCP‐eLi. Charge–discharge profile of eLi||eLi and eLi‐SCP||SCP‐eLi at current density of b) 1 and c) 4 mA cm^−2^ with areal capacity of 1 mAh cm^−2^. d) Schematic of eLi‐SCP||NCM811. e) Rate capability comparison and f) charge–discharge profile of eLi‐SCP||NCM811 and eLi||NCM811 at various scan rate from 1.0 C to 6.0 C. g) Cycle retention analysis and h) charge–discharge profile of eLi‐SCP||NCM811 and eLi||NCM811 during repeated cycle at 4.0 C.

To verify the practical cell configuration with SCP anodeless electrodes (**Figure** **5**), an SCP electrode with electro‐deposited Li (4 mAh cm^−2^, eLi‐SCP) was prepared as the anodeless electrodes for symmetric (eLi‐SCP||eLi‐SCP) and full‐cell configuration (eLi‐SCP||NCM811). As shown in Figure 5a, eLi‐SCP||eLi‐SCP was fabricated with the pair of eLi‐SCP. The long‐term stable operation of eLi‐SCP||eLi‐SCP was observed over 2000 h. Meanwhile, untreated symmetric cells underwent a short‐circuit within 600 h of operation (Figure 5b). This result implies that controlled Li^+^ establishes a uniform solid electrolyte interphase (SEI) layer. This, in turn, induces a low overpotential and stable Li deposition. Moreover, at a current density of 4.0 mA cm^−2^ with an areal capacity of 1.0 mAh cm^−2^, eLi‐SCP||eLi‐SCP operates highly stably without short‐circuit after 250 h (500 cycles, Figure 5c). That is, the overpotential of eLi‐SCP||eLi‐SCP is maintained at 50 mV for 250 h, whereas eLi||eLi shows an increase of overpotential from 50 to 200 mV after 13 h (which results in a short circuit with a dramatic voltage drop after 50 h). The symmetric configuration with graphite and graphene electrodes also shows the rapid increase of overpotential within 30 h due to absence of structural advantages (Figure S11, Supporting Information). The electrochemical performance of eLi‐SCP||eLi‐SCP demonstrates that the stable Li deposition from SCP induces the low polarization and dendrite‐free charging/discharging.

Moreover, the practical performance of eLi‐SCP||NCM811 is evaluated as shown in Figure 5d. For the configuration of eLi‐SCP||NCM811, e‐Li‐SCP prepared with 4.0 mAh cm^−2^ of deposited Li and an NCM811 cathode electrode comprising Super P and PVDF binder in the ratio 90:5:5 are combined. In Figure 5e,f, the stable operation of eLi‐SCP||NCM811 at various scan rates from 1 to 6 C is exhibited (Figure 5e). eLi‐SCP||NCM811 displays high specific capacities of 160, 140, 110 and 70 mAh g^−1^ at 1, 2, 4 and 6 C, respectively. Meanwhile, eLi||NCM811 displays low specific capacity of 141, 118, 79, and 39 mAh g^−1^. The capacity of eLi‐SCP||NCM811 is maintained as the C‐rate increases, with a relatively high capacity retention rate of 43.7% at a relatively high scan rate of 6 C. Meanwhile, the capacity of eLi||NCM811 is maintained with a low capacity retention rate of 27.6% at 6 C. In addition, according to the voltage profile of the increase in C‐rate, eLi‐SCP||NCM811 displays a stable voltage plateau and reliable operation even at a high C‐rate. Meanwhile, eLi||NCM811 exhibits inferior voltage plateau maintenance with a severe voltage drop and low specific capacity (Figure 5f and Figure S12, Supporting Information). The high practical performance is attributed to the anodeless electrodes with dendrite‐free Li growth behavior based on EDL focusing and enhancement of the electro‐osmosis of SCP.^[^ ^13^ ^]^ Consequently, SCP can be an alternative to electrodes for the more effective properties. In Figure 5g,h, eLi‐SCP||NCM811 shows a stable operation without distinct capacity degradation at a scan rate of 4C. A relatively high capacity retention of 99.5% and specific capacity of 110 mAh g^−1^ are observed in eLi‐SCP||NCM811 after 350 cycles, whereas low cycle retention and specific capacity are observed in eLi||NCM811. As shown in Figure 5h, eLi‐SCP||NCM811 displays stable profile maintenance with a low voltage decrease and high specific capacity after 350 cycles. These indicate a remarkable performance durability. Meanwhile, eLi||NCM811 displays a profile variation after 350 cycles with severe voltage variation during charging/discharging. Also, the full‐cell configurations with graphite and graphene electrode show the poor capacity and cycle retention at 4.0 C (Figure S13, Supporting Information). This demonstrates that eLi without SCP displays low Li capability and compatibility with an NCM811 cathode. These results indicate that SCP is compatible with commercially available cathode materials, thereby demonstrating the practical feasibility of high‐performance anode materials for LMBs.

## 3 Conclusion

To summarize, as a Li host material for high capacity and electrochemically stable operation, SCP is recommended for structured carbon materials with narrow space and lithiophilic component sites. Although the general graphite anode displays a sluggish Li storage mechanism owing to the electrochemically limited structure, SCP enables Li^+^ control. This results in a thermodynamically stable Li deposition because of electric field overlapping. The large space structure and residual lithiophilic components promote strong inner electric fields, inducing the dendrite‐free accumulation of Li deposition. Moreover, the self‐smoothing characteristic of SCP can reduce the stripping/deposition overpotential. This would enable a stable operation even with a high current density and large areal capacity. Based on this characterization, Li||SCP|Cu stably performs at a high current density (5 and 10 mA cm^−2^) with long cycle life. The cathode material compatibility of SCP is also remarkable, with a high capacity retention (99.5% during 350 cycles at 4 C) and stable rate capability from 1 C to 6 C. We consider that our strategy would yield the efficient and high performance anode materials. These space structured carbon materials enable a high specific capacity and dendrite‐free Li growth.

## 4 Experimental Section

### Materials

The graphene powders were purchased from Graphene Supermarket. Acetone, 1‐methyl‐2‐pyrrolydinone (NMP), poly(vinylidene fluoride) (PVDF) and electrolyte components such as 1,3‐dioxalne (DOL), dimethoxy ethane (DME), lithium bis(trifluoromethanesulfonyl)imide (LiTFSI) and lithium nitrate (LiNO_3_) for battery evaluation were purchased from Sigma‐Aldrich. The ethyl cyanoacrylate (loctite 401) was purchased from Henkel Adhesive Technologies. Super P conductive carbon black, LiNiCoMnO_2_ (Ni:Co:Mn = 8:1:1), and conductive aluminum foil were purchased from MTI Kora. Lithium foil and copper foil were purchased from Honjo Chemical Corp and UACJ Foil Corp, respectively.

### Preparation of SCP and SCP Electrode

To prepare the SCP, 200 mg of graphene powders was dispersed in 20 mL of acetone. Then, 20 g of ethyl cyanoacrylate was added to the dispersed solution, which was mixed continuously. The dispersed solution was dried in a vacuum oven until the solvent evaporated. Dried samples were crushed by a ball‐milling machine. Then, sample annealing was carried out up to 800 °C for 1 h. Finally, SCP was obtained. To fabricate the SCP electrode, the SCP powder, Super P, and PVDF were mixed in NMP at a mass ratio of 90:5:5 to form slurry. The mixed slurry was cast onto the Cu electrode with an application. The cast Cu electrode was vacuum dried overnight at 60 °C. After drying, the SCP electrode was obtained.

### Characterization of SCP

SEM and TEM images were captured using FEI Magellan XHR 400L and Titan cubed G2 (FEI), respectively. A focused ion beam (Helios Nanolab 450 F1, FEI company) was used for sample preparation to observe the cross‐sections of SCP and graphite by SEM and TEM. In addition, TEM samples for the 3D volume construction were fabricated using a focused ion beam. For the 3D volume construction, TEM images were captured while the sample was rotated at 4° intervals in a range of 80°. The captured images were converted to tomography images using inspect 3D (Thermo Fisher). A 3D volume was generated using the tomography image through Amira software (Thermo Fisher). The width of the gap was measured after meshing.^[^ ^28^, ^29^ ^]^ To determine the pore distribution, Ar adsorption–desorption isotherms were applied using a Quantachrome Instruments Autosorb‐1c apparatus. For the structural comparison of graphite and SCP, XRD patterns were obtained using a SmartLab *θ*–2*θ* diffractometer in the reflectance Bragg–Brentano geometry using a Johansson‐type Ge (111) monochromator with filtered Cu k*α*1 radiation and high‐speed 1D detector (D/teX Ultra). Small‐angle X‐ray scattering (SAXS) was performed using a RIGAKU Nanofix instrument. XPS (K Alpha^+^, Thermo Scientific) analysis was conducted for the chemical state analysis. Raman spectroscopy (Horiba Jobin Yvon) was used to determine the carbon structure. The glass transition temperature and melting temperature of the cyanoacrylate were analyzed using the NETZSCH DSC 214 Polyma. Li^+^ concentration simulations according to the gap spacing of the slits in the electrodes were performed using COMSOL Multiphysics software (COMSOL Group), a CAE module that uses the finite element method. For simulation, the electrochemistry module and the tertiary current distribution model of COMSOL Multiphysics was used.

### Electrochemical Measurement

The electrochemical analyses were conducted using a battery cycler (WBCS3000Le32, Won‐A‐Tech). An electrochemical impedance spectroscope (EIS) was used with VMP3 (Bio‐logic). Li||SCP|Cu was assembled using a 2032‐type coin cell, with an SCP Cu electrode or a bare Cu electrode, and Li foil as the working and counter electrode. The 1 m LiTFSI in DOL/DME solution with 3 wt% LiNO_3_ was used as the electrolyte for all types of batteries. An eLi|SCP electrode was used for the electrochemical evaluation of eLi‐SCP||SCP‐eLi and eLi‐SCP||NCM811. The amount of deposited Li was 4.0 mAh cm^−2^ with a deposition current density of 0.5 mA cm^−2^. To evaluate the eLi‐SCP||NCM811, an NCM811 electrode was prepared using the slurry casting method with Super P, PVDF, and NMP solvent. The cut‐off voltage of each half‐cell was fixed at 1.0 V, and the voltage window of the full cell was applied at 2.8–4.3 V. All types of cells were assembled in an Ar‐filled glove box with O_2_ and H_2_O maintained below 0.1 ppm.

## Conflict of Interest

The authors declare no conflict of interest.

## Supporting information

Supporting Information

## Data Availability

The data that support the findings of this study are available from the corresponding author upon reasonable request.
