# Supplementary material for: Densely Packed Li‐Metal Growth on Anodeless Electrodes by Li+‐Flux Control in Space‐Confined Narrow Gap of Stratified Carbon Pack for High‐Performance Li‐Metal Batteries
Source: Adv Sci (Weinh). 2022 Nov 24;10(3):2205328. doi: 10.1002/advs.202205328 (PMC9875682; doi:10.1002/advs.202205328)
Supplement: Supplementary file 1 — Supporting Information [file ADVS-10-2205328-s001.pdf]

ADVANCED SCIENCE
Open Access

Supporting Information

for *Adv. Sci.*, DOI 10.1002/advs.202205328

Densely Packed Li-Metal Growth on Anodeless Electrodes by Li^{+}-Flux Control in Space-Confined Narrow Gap of Stratified Carbon Pack for High-Performance Li-Metal Batteries

*Jong Ho Won, Woo Hyeong Sim, Donghyoung Kim and Hyung Mo Jeong**

Supporting Information

Densely packed Li-metal growth on anodeless electrodes by Li^+-flux control in space-confined narrow gap of stratified carbon pack for high-performance Li-metal batteries

Jong Ho Won[a,1], Woo Hyeong Sim[b,1], Donghyoung Kim[b] and Hyung Mo Jeong[b,c]*

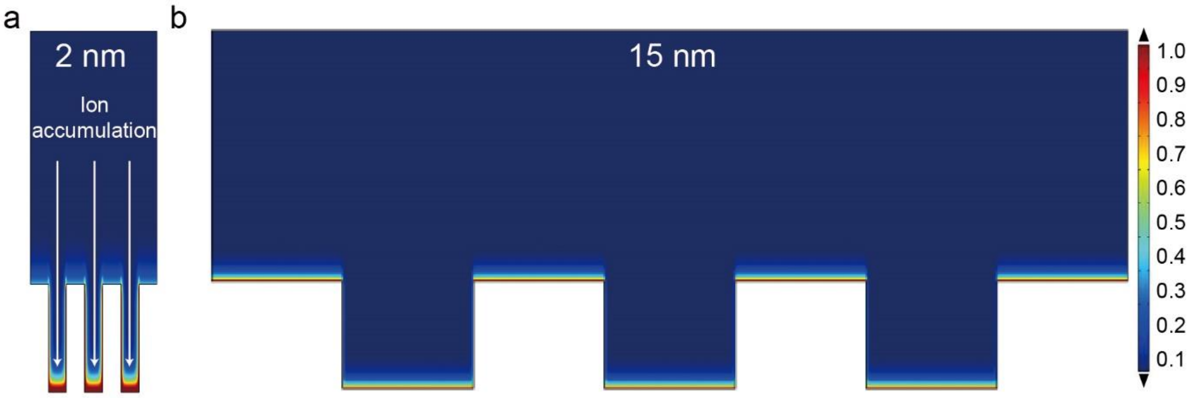

Figure S1. Li^+ concentration distribution according the gap distance of a) 2 nm and b) 15 nm.

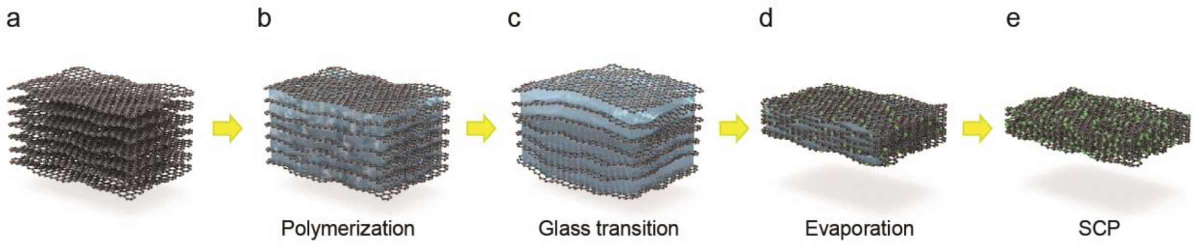

Figure S2. Schematic illustration according to synthesis process of SCP.

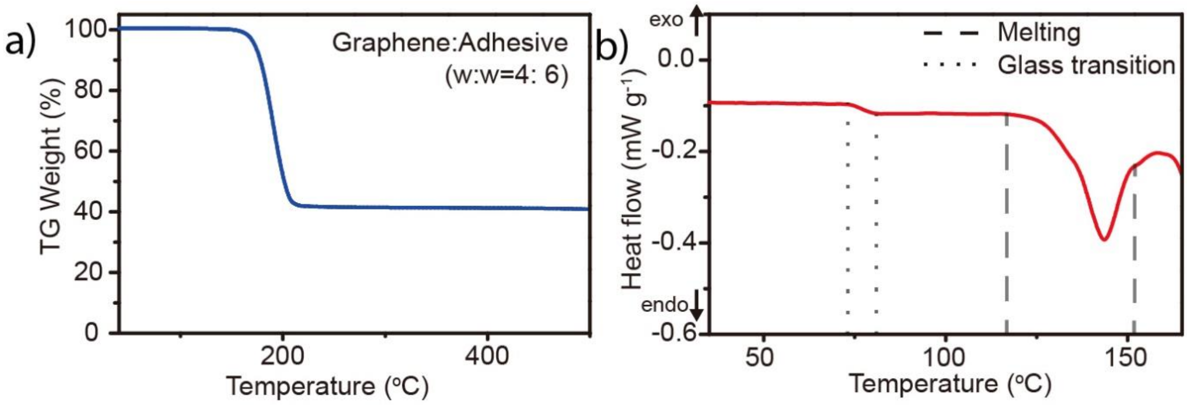

Figure S3. a) The thermogravimetric and b) differential scanning calorimeter analysis data.

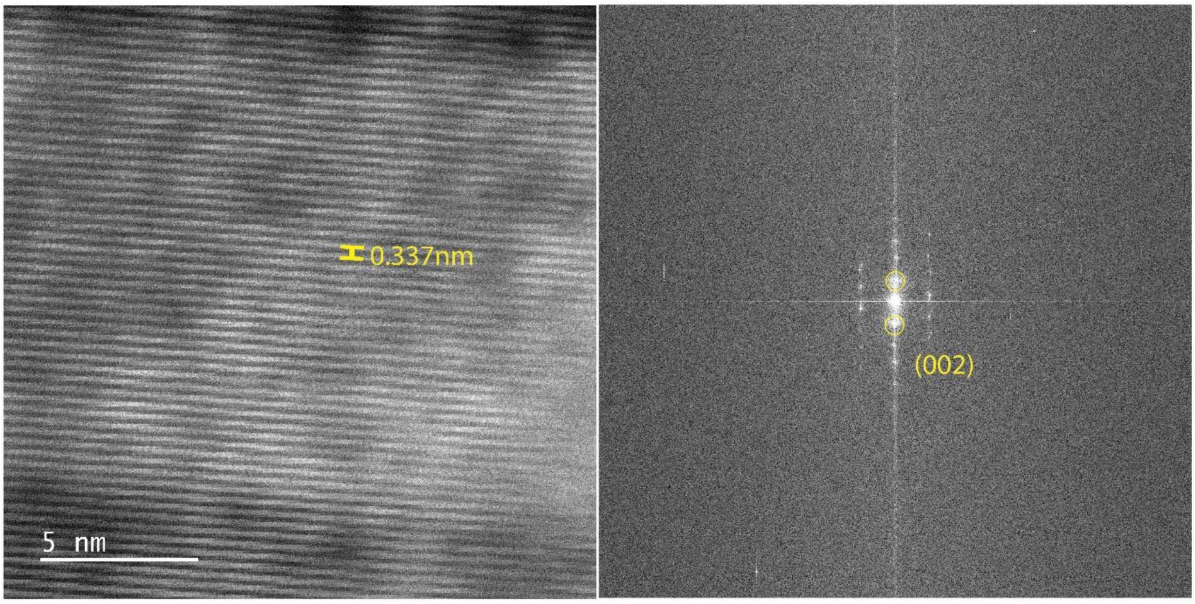

Figure S4. High-magnificaiton TEM images with spacing distance of graphite electrode.

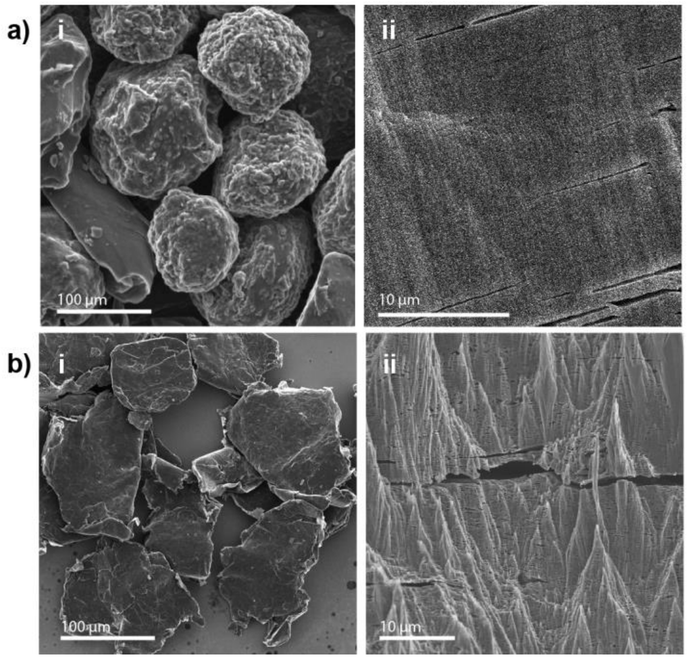

Figure S5. **SEM images of a) graphite and b) SCP**, i) top view image, ii) cross-sectional image.

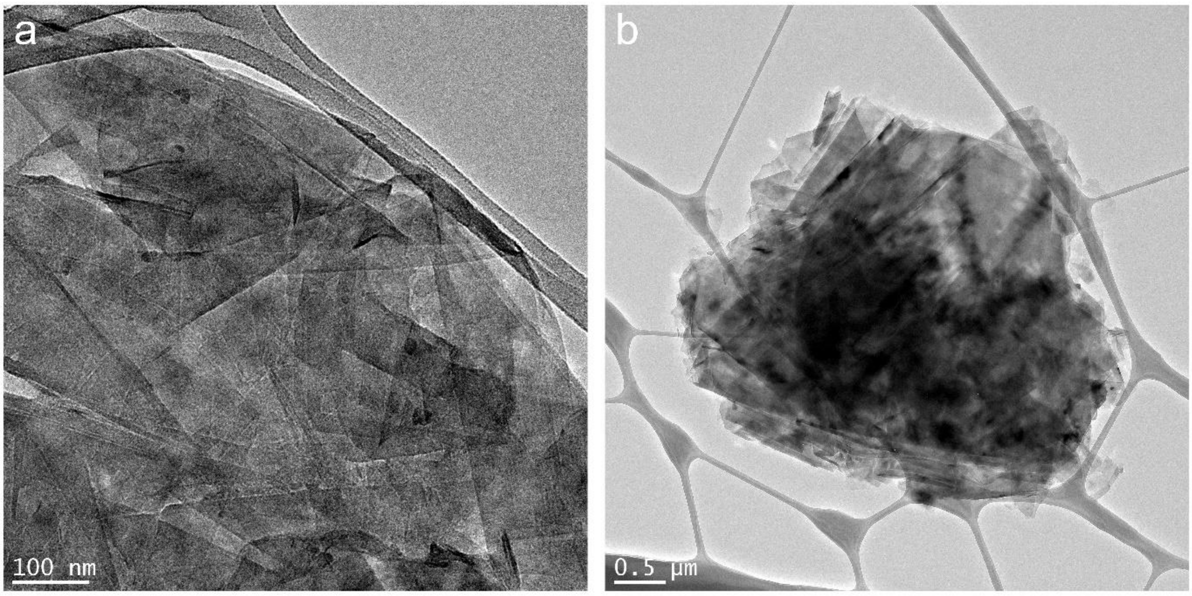

Figure S6. **TEM images of SCP**, a) high magnification and b) low magnification.

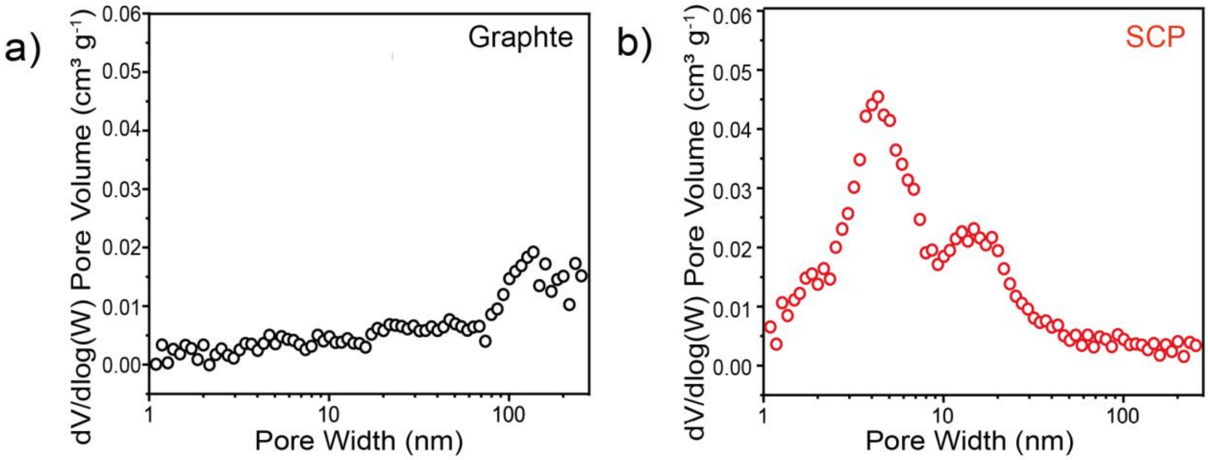

Figure S7. Pore volume and width distribution analysis of graphite and SCP.

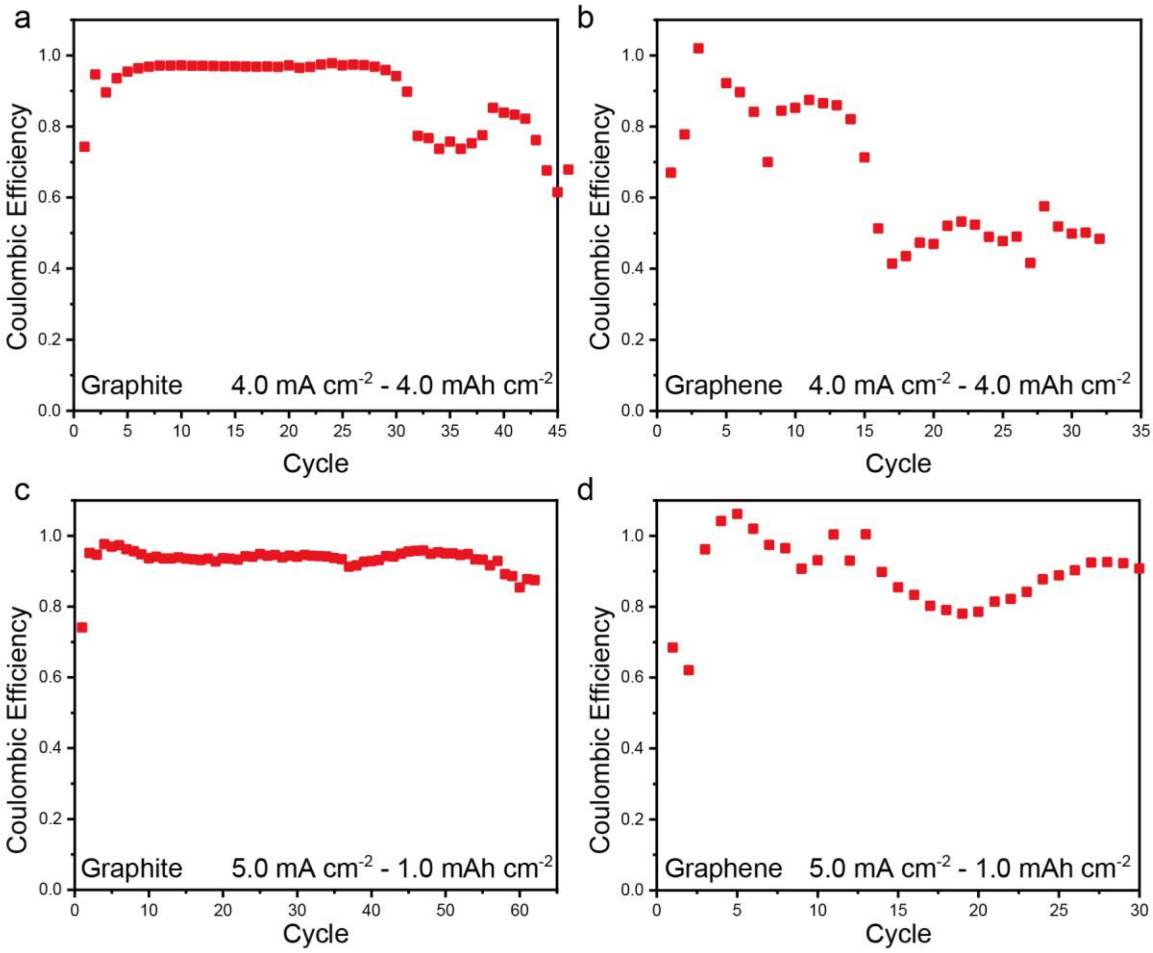

Figure S8. C. E. of half-cell at a condition of 4.0 mA cm^{-2}-4.0 mAh cm^{-2} with a) graphite and b) graphene electrode. C. E. at relatively high current density condition of 5.0 mA cm^{-2}-1.0 mAh cm^{-2} with c) graphite and d) graphene elecrode.

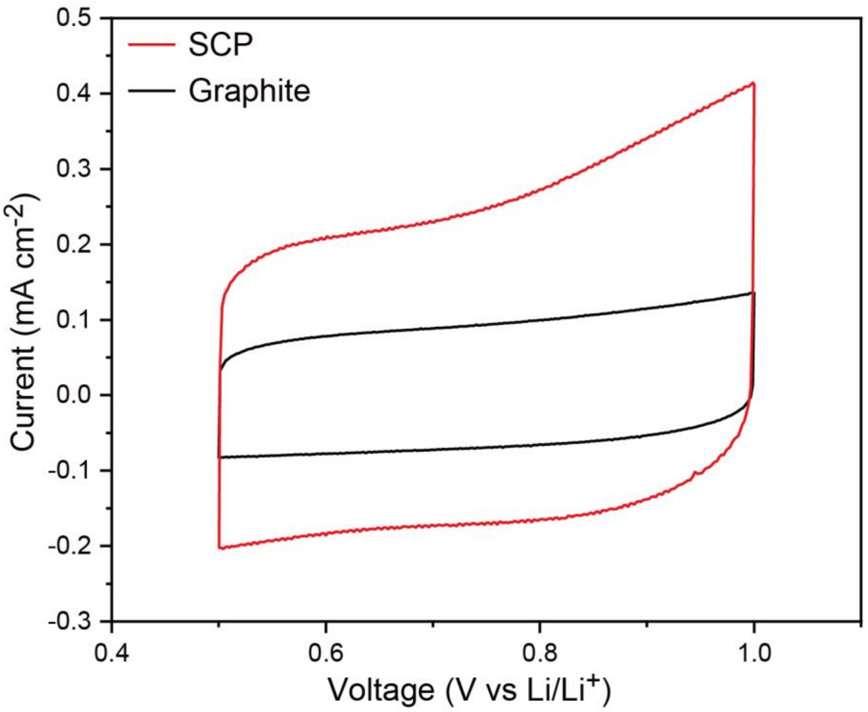

Figure S9. CV curve comparison of SCP and graphite elecrode at scan rate of 50 mV s^{-1}.

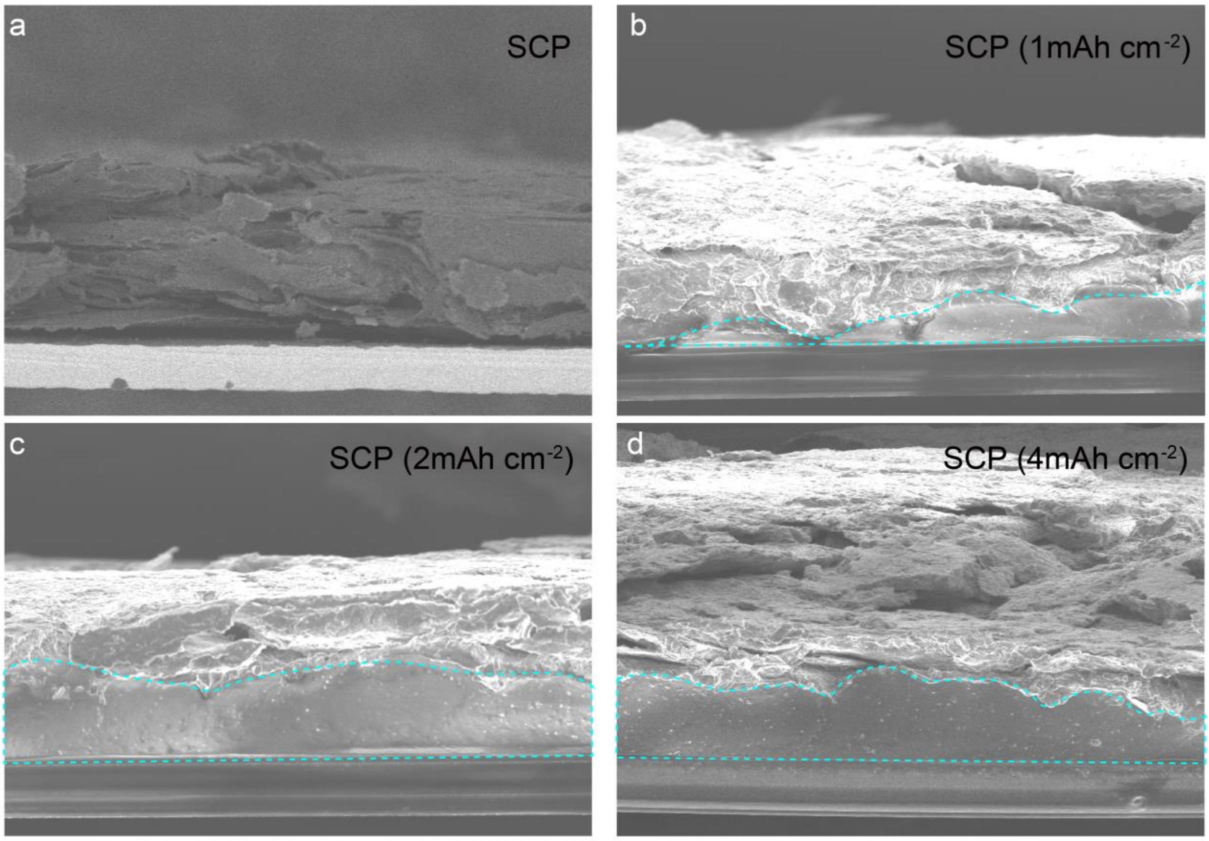

Figure S10. **SEM images of Li deposited SCP electrode according to Li deposition rate,** a) Pre-deposition, b) 1 mAh cm^{-2}, c) 2 mAh cm^{-2}, d) 4 mAh cm^{-2}.

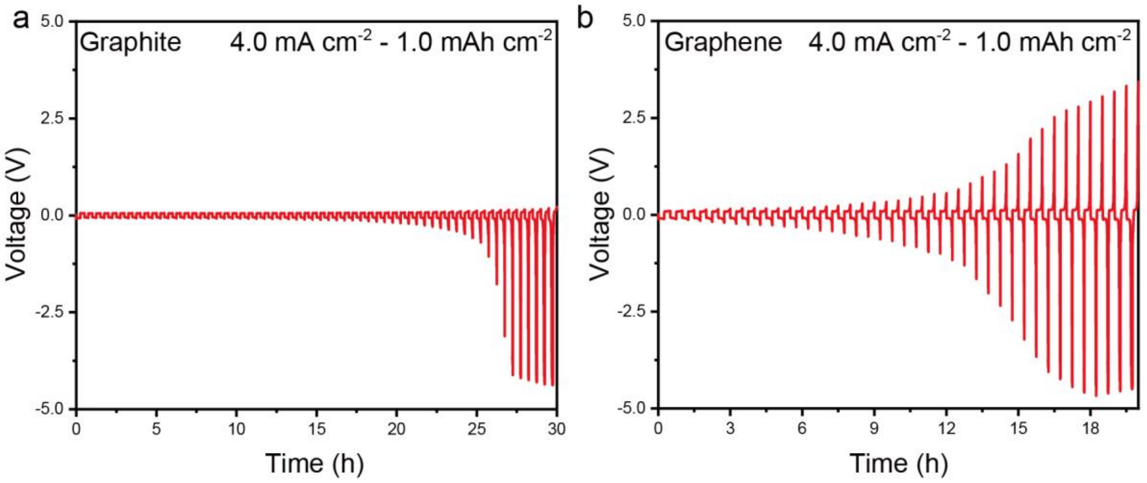

Figure S11. Charge-discharge profile of a) grpahite and b) graphene symmetric cell at current density of 4 mA cm^{-2} with areal capacity of 1 mAh cm^{-2}.

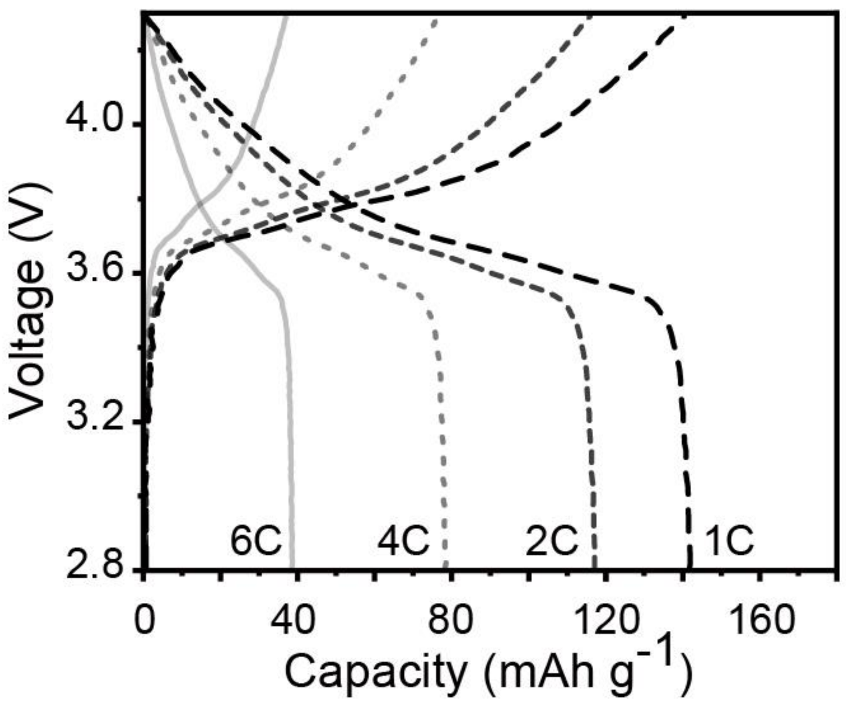

Figure S12. Charge-discharge profile of eLi||NCM811 at various scan rate from 1.0 C to 6.0 C.

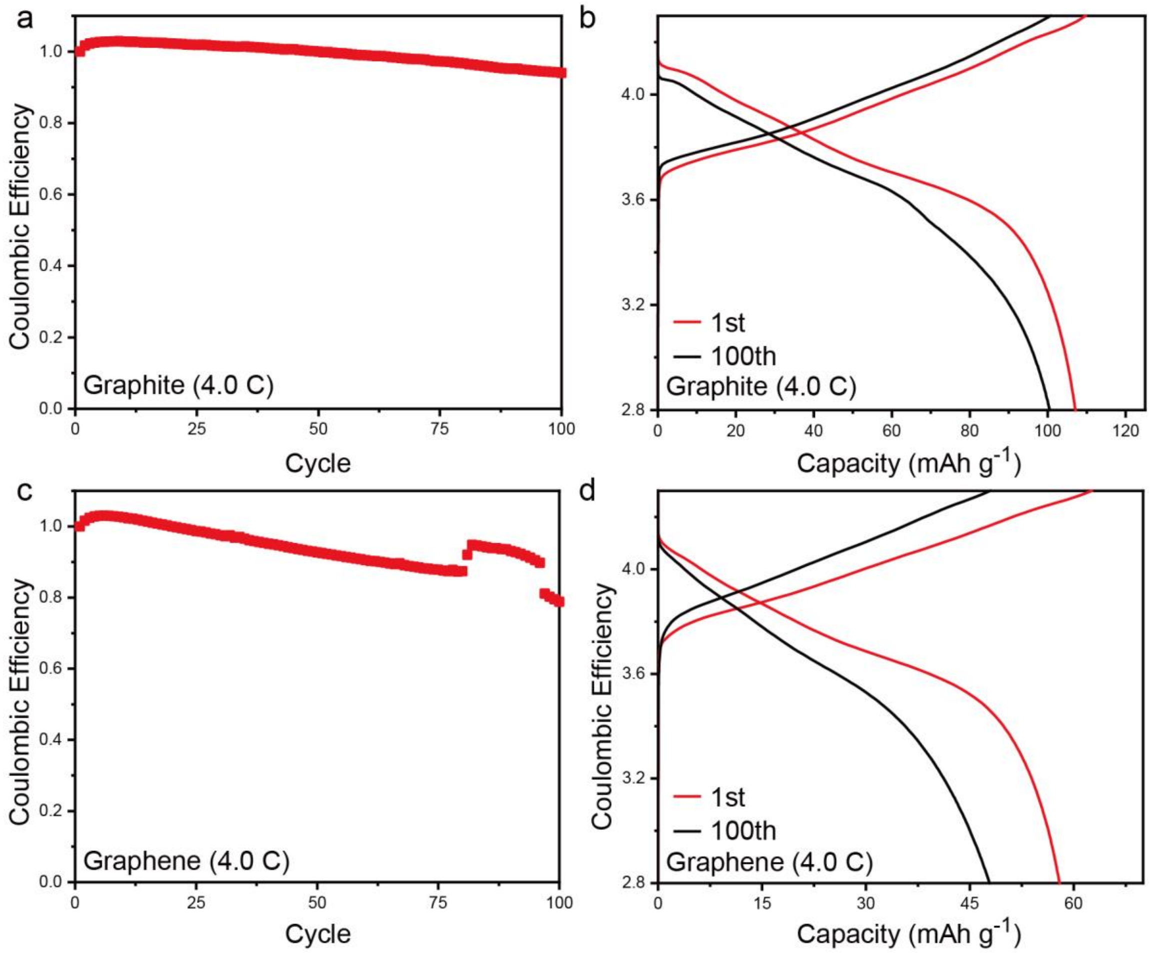

Figure S13. a and b) Cycle retention and charge-discharge profile at 4.0 C rate of full-cell configuration with grpahite and NCM811, c and d) Cycle retention and charge-discharge profile at 4.0 C rate of full-cell configuration with graphene and NCM811.
